# Supplementary material for: Can Research Assessments Themselves Cause Bias in Behaviour Change Trials? A Systematic Review of Evidence from Solomon 4-Group Studies
Source: PLoS One. 2011 Oct 19;6(10):e25223. doi: 10.1371/journal.pone.0025223 (PMC3198466; doi:10.1371/journal.pone.0025223)
Supplement: Box S1 — Hypothetical example. (DOCX) [file pone.0025223.s001.docx]

Can research assessments themselves cause bias in behaviour change trials? A systematic review of evidence from Solomon 4-group studies

Jim McCambridge,^1^ Kaanan Butor-Bhavsar,^1^ John Witton^2^ & Diana Elbourne^3^

| 1: Centre for Research on Drugs & Health Behaviour  Faculty of Public Health & Policy  London School of Hygiene & Tropical Medicine  15-17 Tavistock Place  London WC1H 9SH | 2: National Addiction Centre  Institute of Psychiatry  King’s College London  4 Windsor Walk  London SE5 8AF |
| --- | --- |

3. Department of Medical Statistics

Faculty of Epidemiology & Population Health

London School of Hygiene & Tropical Medicine

Keppel Street

London WC1E 7HT

E-mail: [Jim.McCambridge@lshtm.ac.uk](mailto:Jim.McCambridge@lshtm.ac.uk)

**Abstract**

**Background**: The possible effects of research assessments on participant behaviour have attracted research interest, especially in studies with behavioural interventions and/or outcomes. Assessments may introduce bias in randomised controlled trials by altering receptivity to intervention in experimental groups and differentially impacting on the behaviour of control groups. In a Solomon 4-group design, participants are randomly allocated to one of four arms: (1) assessed experimental group; (2) unassessed experimental group (3) assessed control group; or (4) unassessed control group. This design provides a test of the internal validity of effect sizes obtained in conventional two-group trials by controlling for the effects of baseline assessment, and assessing interactions between the intervention and baseline assessment. The aim of this systematic review is to evaluate evidence from Solomon 4-group studies with behavioural outcomes that baseline research assessments themselves can introduce bias into trials.

**Methodology/Principal Findings**: Electronic databases were searched, supplemented by citation searching. Studies were eligible if they reported appropriately analysed results in peer-reviewed journals and used Solomon 4-group designs in non-laboratory settings with behavioural outcome measures and sample sizes of 20 per group or greater. Ten studies from a range of applied areas were included. There was inconsistent evidence of main effects of assessment, sparse evidence of interactions with behavioural interventions, and a lack of convincing data in relation to the research question for this review.

**Conclusions/Significance:** There were too few high quality completed studies to infer conclusively that biases stemming from baseline research assessments do or do not exist. There is, therefore a need for new rigorous Solomon 4-group studies that are purposively designed to evaluate the potential for research assessments to cause bias in behaviour change trials.

**Introduction**

Behaviour change interventions are increasingly important in public health as awareness of the contribution of behavioural risk factors such as lack of physical activity to an increasingly wide range of health problems grows[1]. Attempts to influence individual behaviour have also gained a new prominence in wider public policy, for example in efforts to combat climate change or terrorism[2]. Randomised controlled trials offer the most rigorous research design to evaluate the effects of behaviour change interventions.

Approximately one hundred years ago control groups were originally introduced in behavioural sciences to address an inferential problem implicit in the use of the single group pre-post design to evaluate intervention effects[3]. It had been observed that pre-testing or assessment itself had effects, which confounded attempts to attribute change over time to intervention, inextricably so with this design. The adoption of non-intervention control groups addressed this problem, which can be termed reactivity, as well as others such as history, maturation and regression to the mean, by making the effects of assessment equivalent between groups[3]. The later advent of randomisation to allocate participants to groups subsequently strengthened the practice of experimentation beyond the laboratory.

Whilst reactivity may be intrinsic to most psychological research [4] and requires particularly careful attention in non-experimental designs, it has been considered much less of a problem in experimental research. So long as reactivity occurs equivalently between-groups, causal inferences about the true effects of interventions are safeguarded by the design of the randomised controlled trial. This conventional “solution” to the problem of reactivity to pre-testing in the two-group trial is not perfect, however, as it does not deal with a possibility first identified by Solomon [3] and later elaborated by Campbell[5], that assessments may interact with interventions to either strengthen or weaken observed effects. In these circumstances 2-group comparisons in trials may produce biased estimates of effects. Solomon thus proposed a 4-group “extension of control group design” in which a further randomisation took place, allocating participants within both the experimental and control groups to be pre-tested or not[3]. As well as offering a means of controlling for assessment effects, the Solomon 4-group design has the capacity to assess interactions between the intervention study conditions and pre-testing. This provides a test of the internal validity of effect sizes obtained in conventional 2-group trials of the effects of behavioural interventions. The possible threat of reactivity to the safety of inference in trials is illustrated with a simple hypothetical example – see Box.

This potential threat applies not only to the adoption of health protective behaviours but equally to the reduction of health compromising behaviours such as smoking cessation. As well as ceiling effects, as described in the hypothetical example, bias could operate in the other direction in situations where there is a synergistic relationship between assessment and intervention. This occurs where research assessment prepares people to be more receptive to intervention than would be the case in the absence of research assessment, for example by prompting contemplation which serves as a preparation for behaviour change.

Conventional trial conduct has previously also been questioned in relation to placebo effects in trials of antidepressant medications. It seems unlikely that drug and placebo effects do not have any multiplicative relationships with each other [6,7]. This work is highly pertinent also because assessment has been identified as a component of the placebo effect in irritable bowl syndrome [8].

The possibility that assessment or measurement may produce bias in trials has been given sustenance by an upsurge in recent health sciences study of assessment reactivity or “mere measurement” effects, the term used within health psychology[9]. Some trials find that earlier research assessments do not influence later outcome data[10,11,12]. Other trials, however, find effects of research assessments on both behavioural and non-behavioural outcomes, both self-reported and objectively ascertained[13,14,15,16,17,18,19,20]. These are usually small in magnitude, which may explain the inconsistency. This recent attention adds to decades of earlier social science research wherein, for example, it is well established that being interviewed on intentions to vote in elections alters the likelihood of actually doing so [21].

The biasing effects of research assessments, if they exist, are likely to be variable across populations, behaviours, interventions and outcomes, as well as the particular assessment methods used. For this reason, an “apples and oranges” evidence synthesis, in which heterogeneity is anticipated at the outset, was judged most appropriate. This could be useful in summarising a broad range of existing relevant information, and in the event that prospective studies are found to be needed, would aid the development of more fine grained hypotheses amenable to testing.

We therefore decided to be as inclusive as possible, incorporating evidence from any Solomon 4-group studies with behavioural outcomes, without regard to particular behaviours, participants, and interventions. The restriction to behavioural outcomes offered the possibility of identifying effects on both objectively ascertained and self-reported measures. Our over-arching research question thus concerned whether any evidence existed that research assessments influenced behaviour in such a way that would indicate bias in behaviour change trials, as identifiable by interactions rather than additive effects observed in Solomon 4-group studies.

**Methods**

In Solomon 4-group studies, participants are randomly allocated to one of four arms: (1) assessed experimental group; (2) unassessed experimental group (3) assessed control group; or (4) unassessed control group. These refer only to baseline assessments and follow-up assessments are undertaken as usual. This design thus provides a test of the internal validity of effect sizes obtained in conventional two-group trials by controlling for the effects of baseline assessment, and assessing interactions between the intervention and baseline assessment.

The early stages of this review were undertaken iteratively, with the final study design decisions resulting from inspection of studies identified in initial searches and more detailed eligibility criteria developed as progress was made. The formal inclusion criteria 1-6 are presented approximately in the sequence that they were applied. Studies must be true applications of the Solomon 4-group design with double randomisation to any intervention and any form of assessment in any population (1) and published in peer-reviewed journals (2); they also needed to have behavioural outcome measures (3) and be undertaken in non-laboratory settings where behaviour was under the autonomous control of study participants (4); also necessary were outcome data for all four groups or an appropriately analysed summary of results (5) with a sample size of 20 per group or more (6). This systematic review was undertaken without a published protocol.

Electronic database searches without date restrictions were undertaken in Web of Knowledge, PsychInfo, CINAHL Plus with full text, INSPEC, ERIC, Web of Science, Medline, Pubmed, Cochrane Central Register of Controlled trials, EMBASE, BIOSIS Previews, Sociological Abstracts, National Criminal Justice Reference Service Abstracts (NCJRS), Social Services Abstracts, Linguistics and Language Behaviour Abstracts (LLBA), the International Bibliography of the Social Sciences (IBSS), Biomed Central, APPI Journals, British Nursing Index, ADOLEC, AgeInfo, Allied and Complementary Medicine Database (AMED), Medline inc, Social Policy and Practice, British Humanities Index, Applied Social Sciences Index and Abstracts (ASSIA), and PsychArticles. The basic search strategy, for example as used in Medline, was Topic=(solomon 4) OR Topic=(solomon four). This was supplemented where it was possible with NOT Author=(Solomon) and NOT Topic=(island*).

After screening for relevance by title and abstract all subsequent inclusion/exclusion decisions were made jointly by two authors, with a third opinion occasionally sought for irreconcilable differences of opinion. After the initial searches an update on 24/08/10 yielded no additional inclusions, nor did contacts with experts. Data extraction from included studies was undertaken by two authors from published reports with a dedicated form and without any contact with their authors. This comprises the data presented in Tables 1-4 and the accompanying text including additional quantitative data, which also addresses relevant sources of bias. Given the nature of our research question and the heterogeneity of included studies this tabular and narrative presentation was preferred. This decision not to undertake a meta analytic synthesis was made after the dataset had been finalised. Risk of bias across studies is considered in the discussion section in light of obtained findings.

**Results**

Ten studies were eligible for inclusion in this review [22,23,24,25,26,27,28,29,30,31] – see Figure for a summary of the study selection process and Table 1 for details of included studies. The majority (n=6) of these studies took place in schools and were concerned with the prevention of health compromising behaviours among children. The four studies with adults evaluated health promotion interventions. The two smallest studies also had the shortest periods of follow-up study. The four adult studies comprised similar sample sizes and follow-up intervals (see Table 1). The baseline research assessments were with questionnaires in all cases bar two, in which interviews took place[22,23].

There was somewhat more consistent evidence of intervention main effects in adult than in the school-based studies and greater evidence of assessment main effects on non-behavioural as compared to behavioural outcomes – see Table 2. There was weak evidence only of interaction effects between interventions and assessments on behavioural outcomes overall. The quality of reporting was variable across these studies.

*Studies with adults*

Dignan and colleagues conducted two similar studies evaluating the effects of health education on cervical cancer screening attendance with Solomon 4 group designs in two different Native American tribes[22,23]. In these studies face-to-face interviews delivered by members of the relevant tribes were the research assessments evaluated. These were studied alongside the health education intervention as there are pervasive beliefs in Native American cultures that health is sacred and talking about related beliefs or behaviours is injurious to health[23]. The timeframe of the behavioural outcome measure was 12 months, whilst the follow-up interval was 6 months in both studies.

In the first of these studies[22], the main assessment effect on screening attendance just missed the conventional threshold for statistical significance (OR=1.65 [0.97-2.81]) and the reported interaction test result on screening attendance was not statistically significant (OR=0.88 [0.38-2.03]). It seems likely, however, that this measure of effect applies to the comparison between the group who were both assessed and had the intervention and the group who had neither. In the raw data there is an indication of an interaction – see Table 3. Differences apparently due to the main effects of assessments are small and inconsistent, whilst the effect of the intervention appears to be approximately twice as large among those who have not been assessed as compared to those who have, suggesting that the observed intervention effect depends upon whether assessment has taken place[22].

In the second of these studies there are no clear effects of the assessment interview nor interactions with intervention, which was again found to be effective in promoting screening attendance in a logistic regression model[23]. These data are also presented in Table 3 for comparison purposes.

Lusk and colleagues provide ANOVA results showing an intervention effect, and no pre-testing main effect nor interaction with intervention[24]. There are no more detailed data available for the purposes of this study.

VanSluijs and colleagues [25] provide evidence of assessment main effects with the proportions meeting guideline levels of physical activity higher among those assessed twice with the 13 page booklet in both unadjusted and adjusted analyses (OR=1.70 [1.14-2.54]). It should be remembered that the control condition in this study were also exposed to intervention, albeit less intensive than the principal intervention being evaluated. VanSluijs and colleagues state that “no effect modification for randomization to control or intervention condition was observed” without reporting any additional data[25].

*Secondary school studies*

Kvalem and colleagues [27] in Norway investigated the effects of training for peer sex education on condom use at most recent intercourse on those trained and not on those to whom the sex education was to be delivered. An attempt was made to account for clustering in classes in this study by adding a class attribute variable in the outcome model; this made little difference to outcomes. Unlike all other included studies, an interaction effect is presented among the sub-group of 403 participants who had had their first intercourse prior to the study and who provided follow-up data after 6 months – see Table 4. All three other conditions are found to be distinct from the reference group of those who were pretested and received the intervention (ORs 0.31, 0.42 and 0.41, p=0.005 or less for each comparison). The interpretation given by the authors to these data is that the effect of the training depends upon the prior completion of the questionnaire “to give students an opportunity for greater reflection on their own sexual behaviour.”[27] After 12 months a somewhat similar pattern of results was observed with odds ratios closer to 1 and not statistically different from the reference group of those both pre-tested and received intervention (ORs 0.61 [p=0.24], 0.57 [p=0.09] and 0.73 [p=0.35]) among the 355 providing follow-up data. Smaller numbers were randomised to intervention groups in this study and there appears to be differential attrition by group. Condom use at first intercourse among those who had not had sex prior to the study was also investigated, though outcome data for all 4 groups were not presented[27].

A subsequent Norwegian study was published by Traeen[28]. This has significant reporting problems in connection with the aims of this study due to, for example, not consistently reporting outcome data for all 4 groups. In one instance, data suggest the presence of an interaction effect on use of contraception during most recent intercourse.[28] There is a difference of 7% favouring the intervention among those who were assessed (64/107 [59%] compared to 24/45 [52%]) and a difference of 10% favouring the non-intervention control condition among those who were not assessed (60/105 [57%] compared to 43/64 [67%]). Differences among the proportions are not tested and odds ratios presented in a multivariate logistic regression model were not statistically significant[28]. Differences in sample size among the study conditions after allowing for higher allocation to intervention conditions give scope for concern about the possible effects of attrition bias. The Discussion section begins with the statement that “The results from this study have shown a significant effect of the intervention in interaction with the pre-test on use of contraception during the first intercourse in adolescents who made their coital debut in the period from the pre-test to the first post-test.”[28] This statement appears to depend on data not resulting from four-group analyses. As with the previous study, the quantitative data reported above are obtained among a sub-group of those randomised rather than in the study population as a whole.

Inspection of the data in the study by Duryea [26] suggests it is unlikely that there were any interactions between assessment and intervention for either outcome even were this small study to have been very much larger.

*Primary school studies*

Two of the three primary school studies were based on the same cohort which was the largest in this literature. Campanelli and colleagues in evaluating alcohol prevention effects [29] found pre-testing main effects on 1 of 3 alcohol misuse variables in the more appropriate adjusted analyses taking account of clustering in schools, and also in a second alcohol misuse variable in less rigorous unadjusted analyses. In both cases there were higher scores indicating greater alcohol misuse among those who had not been pre-tested, indicating the possibility of a small beneficial effect of the 60-item questionnaire. They found no statistically significant interaction effects on relevant behavioural outcomes, with only the possibility of a weak trend in this direction being discernible in the case of alcohol use frequency[29]. Probably because of these initial findings, much less attention is paid to assessment effects in the later follow-up of this sample by Shope et al.[30] Among 5^th^ grade students providing follow-up data at all 3 intervals, the pre-tested intervention group had somewhat lower scores than other groups (0.37 [0.91] compared to 0.49 [1.05], 0.51 [1.07] and 0.55 [1.07] on a combined outcome measure at the same first follow-up as previously reported[30].

The other primary school study by Freeman [31] provides little data useful here by virtue of its design (see Table 1*). A priori* the possibility of an interaction between this an assessment measure of academic ability and a creative drama intervention to address problem behaviour seems unlikely. Neither intervention nor assessment effects were found in this study, in which the study population was young (school grades 3 and 4).

**Discussion**

This systematic review was primarily designed to discover whether there was evidence of interactions in existing Solomon 4 group studies with behavioural outcomes. Any such evidence could be indicative of research assessments causing bias in conventional behaviour change trials. The principal finding, therefore, is that there is meagre evidence of interactions in existing studies. Whilst there are many applications in laboratory-based psychology and in classrooms for educational research, the Solomon 4 group design has not been widely used in social and health sciences in studies with behavioural outcomes. Existing applications are highly heterogeneous and meta analytic synthesis of their main findings was judged inadvisable.

It is worth considering why there have been so few Solomon 4 group studies. The design may appear somewhat complex and there are studies which have failed to implement it successfully, particularly due to analytic problems [32]. Randomisation itself is not, however, achieved with any more difficulty. The design may also be considered to be relatively expensive in terms of statistical power and required study resources. It has thus been used only in situations where there has been a particular concern about assessment effects interfering with study outcomes. The particular need to reliably estimate small behavioural intervention effects that can be widely obtained in populations is arguably a quite recent concern, or at least it is now being taken more seriously than was the case previously. There is also now more careful attention to research assessment reactivity and possible impacts on other forms of bias [33] as well as on research participation effects in trials more broadly [34].

Of the two studies providing any evidence of interactions in sub-groups, in one case data were clearly appropriately analysed[27], and in the other case this was unlikely[28]. Reporting problems are apparent and although methodological quality was not formally assessed here, both sets of findings are vulnerable to various biases. There were main effects on self –reported behavioural outcomes clearly attributable to research assessments in two other studies[25,29]. There were also main effects of research assessments on non-behavioural self-reported outcomes in both of these studies and in two additional studies, on knowledge [26] and intentions[23], with an interaction effect also in the latter case.

It should be expected that interactions, if they exist, would be variable across populations and behaviours and depend upon the precise features of the assessment and intervention methods. Similarities between the contents of research assessments and interventions and their component parts provide *a priori* grounds for concern about the potential for bias. For example, pedometers may be used both as an intervention component and in research assessment in studies of interventions promoting walking [35]. Any evidence of interaction, however small the effects may be, entails bias in estimates of intervention effectiveness, and thus deserves to be investigated.

There are perhaps two different types of research question that may be asked about this phenomenon. 1) To ask, as we have done here, *can* research assessments themselves cause bias in behaviour change trials? This is analogous to designing an efficacy or explanatory trial to answer this question. One would seek conditions in which the purported effect was most likely to be found, perhaps selecting particular behaviours and study populations, research assessment and intervention materials, judged favourable to assessment reactivity by some criteria, in circumstances lending themselves to reliable quantification. 2) Alternatively, one could ask, *do* research assessments themselves cause bias in behaviour change trials? Are there problems with conventional practice in behaviour change trials that we don’t yet know about? This is analogous to designing an effectiveness or pragmatic trial to answer this question. Presumably, one would choose typical research assessment contents and well evaluated interventions for behaviours of clear population health importance. This latter type of question is the more important question to ask, though arguably logic first requires an affirmative answer to the first question.

This systematic review has been designed in various ways which engender confidence in the reported findings and attention is also warranted to its limitations. Restricting inclusion to peer reviewed studies should not have biased findings to be more or less likely to produce evidence of interactions. Requiring reporting of outcome data for all 4 groups served to exclude studies whose findings were difficult to interpret. Arguably the identification of interactions only becomes possible once appropriate analyses have been successfully implemented. There were many incorrect analyses used in excluded studies and the most appropriate statistical methods for Solomon 4 group studies were determined some decades after the introduction of the design [32].

Excluding studies with small sample sizes is an unusual decision, though there are reasons to be concerned about the influence of small study effects in reviews [36,37]. As well as publication bias, smaller studies are more vulnerable to other forms of bias. This decision was taken after initial scoping indicated a number of unusual studies which were difficult to describe well and whose contribution was judged likely not to be very helpful. The threshold was set somewhat arbitrarily at a low level only to exclude very small studies (n=20 per group, total n=80). By way of example, Lawson and Frankish [38] started with a total n=40 which subsequently attrited to n=16. It must be recognised, however, that there are otherwise well conducted Solomon 4 group studies with very small sample sizes that have been excluded (for example,[39,40]) and that their inclusion could be valuable if subsequent reviews are able to undertake quantitative syntheses when the literature is better developed.

Inclusion in this review was limited to studies with behavioural outcome measures. It transpired that there were no studies with observed or otherwise objectively ascertained outcomes. The exclusion of cognitive, affective and other types of psychological outcomes should be carefully considered. Prior work in this area has found assessment effects to be larger in these other areas than on behavioural outcomes [41,42]. Where such data are important outcomes in behaviour change trials they may be biased by the interactions between assessment and intervention effects. It seems likely, therefore, that there exist Solomon 4 group studies which can provide data on the possibility of interactions that lie beyond the limits of this review. Solomon 4 group studies may be particularly valuable for studies with patient reported outcomes, for example [43], and this may be a fruitful avenue for further research. Given the nature of the target study design, it is unlikely that we will have missed studies within our inclusion criteria that should have been included unless they have not used the Solomon label. This is indeed possible, though not being aware of any such studies makes it impossible to gauge how likely this is.

There are too few completed rigorous studies to infer that the interactions targeted for study either simply do or do not exist. Conduct of this study has, however, advanced hypotheses about the nature of the possible effects to be evaluated in a number of ways. Situations in which both interventions and assessments may be expected to exert main effects upon behaviour are conducive to tests of their possible interactions. Meta-analytic data providing preliminary evidence of assessment effects in the alcohol field have recently been published [44]. This extends a history of earlier attention to these issues in that field which has been based upon the idea that assessment enhances capacity for self-regulation [45]. Although children may be more susceptible to assessment effects, they may less receptive to dedicated interventions, and adult populations may be preferable for these reasons. Sample sizes should be as large as possible. Synergistic effects as well as ceiling effects are both plausible and will likely depend upon the particular behaviour selected for study and motivations and more broadly the relationship of the study population to the behaviour. The main conclusion is that this review demonstrates the need for new Solomon 4-group studies that are purposively designed to evaluate the potential for research assessments themselves to cause bias in behaviour change trials.

**Box: Hypothetical example**

**Figure: PRISMA 2009 Flow Diagram**

**References**

**1. Lopez AD, Mathers, C. D., Ezzati, M., Jamison, D. T. and Murray, C. J. L. (2006) Global and regional burden of disease and risk factors, 2001: Systematic analysis of population health data. Lancet 367: 1747-1757.**

**2. Institute for Government & UK Government Cabinet Office (2010) MINDSPACE: Influencing behaviour through public policy.**

**3. Solomon RL (1949) An extension of control group design. Psychol Bull 46: 137-150.**

**4. Fox MC, Ericsson KA, Best R (2011) Do procedures for verbal reporting of thinking have to be reactive? A meta-analysis and recommendations for best reporting methods. Psychol Bull 137: 316-344.**

**5. Campbell DT (1957) Factors relevant to the validity of experiments in social settings. Psychol Bull 54: 297-312.**

**6. Kirsch I (2000) Are drug and placebo effects in depression additive? Biol Psychiatry 47: 733-735.**

**7. Kirsch I, Deacon BJ, Huedo-Medina TB, Scoboria A, Moore TJ, et al. (2008) Initial severity and antidepressant benefits: a meta-analysis of data submitted to the Food and Drug Administration. PLoS Med 5: e45.**

**8. Kaptchuk TJ, Kelley JM, Conboy LA, Davis RB, Kerr CE, et al. (2008) Components of placebo effect: randomised controlled trial in patients with irritable bowel syndrome. BMJ 336: 999-1003.**

**9. McCambridge J (2009) [Commentary] Research assessments: instruments of bias and brief interventions of the future? Addiction 104: 1311-1312.**

**10. Daeppen JB, Gaume J, Bady P, Yersin B, Calmes JM, et al. (2007) Brief alcohol intervention and alcohol assessment do not influence alcohol use in injured patients treated in the emergency department: a randomized controlled clinical trial. Addiction 102: 1224 - 1233.**

**11. del Junco DJ, Vernon SW, Coan SP, Tiro JA, Bastian LA, et al. (2008) Promoting regular mammography screening I. A systematic assessment of validity in a randomized trial. J Natl Cancer Inst 100: 333-346.**

**12. Cherpitel CJ, Korcha RA, Moskalewicz J, Swiatkiewicz G, Ye Y, et al. (2010) Screening, brief intervention, and referral to treatment (SBIRT): 12-month outcomes of a randomized controlled clinical trial in a Polish emergency department. Alcohol Clin Exp Res 34: 1922-1928.**

**13. Kypri K, Langley JD, Saunders JB, Cashell-Smith ML (2007) Assessment may conceal therapeutic benefit: findings from a randomized controlled trial for hazardous drinking. Addiction 102: 62-70.**

**14. Carey KB, Carey MP, Maisto SA, Henson JM (2006) Brief motivational interventions for heavy college drinkers: A randomized controlled trial. Journal of Consulting and Clinical Psychology 74: 943 - 954.**

**15. Clifford PR, Maisto SA, Davis CM (2007) Alcohol treatment research assessment exposure subject reactivity effects part I. Alcohol use and related consequences. Journal of Studies on Alcohol and Drugs 68: 519 - 528.**

**16. Godin G, Sheeran P, Conner M, Germain M (2008) Asking questions changes behavior: mere measurement effects on frequency of blood donation. Health Psychol 27: 179-184.**

**17. McCarney R, Warner J, Iliffe S, van Haselen R, Griffin M, et al. (2007) The Hawthorne Effect: a randomised, controlled trial. BMC Med Res Methodol 7: 30.**

**18. Sandberg T, Conner M (2009) A mere measurement effect for anticipated regret: impacts on cervical screening attendance. Br J Soc Psychol 48: 221-236.**

**19. Walters ST, Vader AM, Harris TR, Jouriles EN (2009) Reactivity to alcohol assessment measures: an experimental test. Addiction 104: 1305-1310.**

**20. McCambridge J, Day, M. (2008) Randomized controlled trial of the effects of completing the Alcohol Use Disorders Identification Test questionnaire on self-reported hazardous drinking. Addiction 103: 241-248.**

**21. Kraut RE, Mcconahay.Jb (1973) How Being Interviewed Affects Voting - Experiment. Public Opinion Quarterly 37: 398-406.**

**22. Dignan M, Michielutte R, Blinson K, Wells HB, Case LD, et al. (1996) Effectiveness of health education to increase screening for cervical cancer among eastern-band Cherokee Indian women in North Carolina. J Natl Cancer Inst 88: 1670-1676.**

**23. Dignan MB, Michielutte R, Wells HB, Sharp P, Blinson K, et al. (1998) Health education to increase screening for cervical cancer among Lumbee Indian women in North Carolina. Health Educ Res 13: 545-556.**

**24. Lusk SL, Hong OS, Ronis DL, Eakin BL, Kerr MJ, et al. (1999) Effectiveness of an intervention to increase construction workers' use of hearing protection. Human Factors 41: 487-494.**

**25. van Sluijs EM, van Poppel MN, Twisk JW, van Mechelen W (2006) Physical activity measurements affected participants' behavior in a randomized controlled trial. J Clin Epidemiol 59: 404-411.**

**26. Duryea EJ (1983) Utilizing tenets of inoculation theory to develop and evaluate a preventive alcohol education intervention. J Sch Health 53: 250-256.**

**27. Kvalem IL, Sundet JM, Rivo KI, Eilertsen DA, Bakketeig LS (1996) The effect of sex education on adolescents' use of condoms: applying the Solomon four-group design. Health Educ Q 23: 34-47.**

**28. Traeen B (2003) Effect of an intervention to prevent unwanted pregnancy in adolescents. A randomized, prospective study from Nordland County, Norway, 1999-2001. Journal of Community & Applied Social Psychology 13: 207-223.**

**29. Campanelli PC, Dielman TE, Shope JT, Butchart AT, Renner DS (1989) Pretest and treatment effects in an elementary school-based alcohol misuse prevention program. Health Educ Q 16: 113-130.**

**30. Shope JT, Dielman TE, Butchart AT, Campanelli PC, Kloska DD (1992) An elementary school-based alcohol misuse prevention program: a follow-up evaluation. J Stud Alcohol 53: 106-121.**

**31. Freeman GD, Sullivan K, Fulton CR (2003) Effects of creative drama on self-concept, social skills, and problem behavior. Journal of Educational Research 96: 131-138.**

**32. Braver MCW, Braver, S.L. (1988) Statistical treatment of the Solomon four-group design: A meta-analytic approach. Psychological Bulletin 104: 150-154.**

**33. McCambridge J, Kalaitzaki, E., White, I.R., Khadjesari, Z., Murray, E., Linke, S., Thompson, S.G., Godfrey, C., Wallace, P. (2011) Can differences in the length or relevance of questionnaires impact upon attrition in online trials? A randomised controlled trial. . Journal of Medical Internet Research in press.**

**34. Kypri K, McCambridge J, Wilson A, Attia J, Sheeran P, et al. (2011) Effects of Study Design and Allocation on participant behaviour - ESDA: study protocol for a randomized controlled trial. Trials 12: 42.**

**35. Clemes SA, Parker RA (2009) Increasing our understanding of reactivity to pedometers in adults. Med Sci Sports Exerc 41: 674-680.**

**36. Nuesch E, Trelle S, Reichenbach S, Rutjes AW, Tschannen B, et al. (2010) Small study effects in meta-analyses of osteoarthritis trials: meta-epidemiological study. BMJ 341: c3515.**

**37. Coyne JC, Thombs BD, Hagedoorn M (2010) Ain't necessarily so: review and critique of recent meta-analyses of behavioral medicine interventions in health psychology. Health Psychol 29: 107-116.**

**38. Lawson DM, Frankish CJ (1982) Anticipated Versus Unanticipated Fitness Assessment and Dropout Following Exercise Prescription. Journal of Behavioral Assessment 4: 287-297.**

**39. Mungas DM, Walters HA (1979) Pretesting Effects in the Evaluation of Social Skills Training. Journal of Consulting and Clinical Psychology 47: 216-218.**

**40. Spence JC, Burgess J, Rodgers W, Murray T (2009) Effect of pretesting on intentions and behaviour: A pedometer and walking intervention. Psychology & Health 24: 777-789.**

**41. Willson VL, Putnam, R.R. (1982) A meta-analysis of pretest sensitization effects in experiemntal design. American Educational Research Journal 19: 249-258.**

**42. French DP, Sutton S (2010) Reactivity of measurement in health psychology: how much of a problem is it? What can be done about it? Br J Health Psychol 15: 453-468.**

**43. Braeken AP, Lechner L, van Gils FC, Houben RM, Eekers D, et al. (2009) The effectiveness of the Screening Inventory of Psychosocial Problems (SIPP) in cancer patients treated with radiotherapy: design of a cluster randomised controlled trial. BMC Cancer 9: 177.**

**44. McCambridge J, Kypri. K. (2011) Can simply answering research questions change behaviour? Systematic review and meta analyses of brief alcohol intervention trials. PLoS ONE in press.**

**45. Clifford PR, Maisto SA (2000) Subject reactivity effects and alcohol treatment outcome research. J Stud Alcohol 61: 787-793.**
